# Insulin prevents and reverts simvastatin-induced toxicity in C2C12 skeletal muscle cells

**DOI:** 10.1038/s41598-019-43938-5

**Published:** 2019-05-15

**Authors:** Gerda M. Sanvee, Jamal Bouitbir, Stephan Krähenbühl

**Affiliations:** 1 Division of Clinical Pharmacology & Toxicology, University Hospital, Basel, Switzerland; 2 Department of Biomedicine, University of Basel, Basel, Switzerland; 3 Swiss Centre for Applied Human Toxicology (SCAHT), Basel, Switzerland

**Keywords:** Molecular biology, Molecular medicine

## Abstract

Simvastatin is an inhibitor of the 3-hydroxy-3-methylglutaryl-CoA reductase used for decreasing low density lipoprotein (LDL)-cholesterol in patients. It is well-tolerated but can cause myopathy. Our aims were to enlarge our knowledge regarding mechanisms and effects of insulin on simvastatin-associated myotoxicity in C2C12 myotubes. Simvastatin (10 µM) reduced membrane integrity and ATP content in myotubes treated for 24 hours, which could be prevented and partially reversed concentration- and time-dependently by insulin. Furthermore, simvastatin impaired the phosphorylation of Akt (Protein Kinase B) mainly at Ser473 and less at Thr308, indicating impaired activity of the mammalian Target of Rapamycin Complex 2 (mTORC2). Impaired activation of Akt increased mRNA expression of the muscle atrophy F-Box (MAFbx), decreased activation of the mammalian Target of Rapamycin Complex 1 (mTORC1) and stimulated apoptosis by impairing the Ser9 phosphorylation of glycogen synthase kinase 3β. Decreased phosphorylation of Akt at both phosphorylation sites and of downstream substrates as well as apoptosis were prevented concentration-dependently by insulin. In addition, simvastatin caused accumulation of the insulin receptor β-chain in the endoplasmic reticulum (ER) and increased cleavage of procaspase-12, indicating ER stress. Insulin reduced the expression of the insulin receptor β-chain but increased procaspase-12 activation in the presence of simvastatin. In conclusion, simvastatin impaired activation of Akt Ser473 most likely as a consequence of reduced activity of mTORC2. Insulin could prevent the effects of simvastatin on the insulin signaling pathway and on apoptosis, but not on the endoplasmic reticulum (ER) stress induction.

## Introduction

Statins or 3-hydroxy-3-methylglutaryl-CoA reductase inhibitors represent a drug class, which is used widely in patients with cardiovascular diseases in order to lower LDL-cholesterol^1^. Treatment with statins in such patients has been demonstrated to decrease morbidity and mortality in large studies^2^. Statins are considered to be safe, but up to 30% of the patients treated with these drugs can develop signs and/or symptoms of muscle injury^3^. Muscle injury in such patients includes elevated activity of serum creatine kinase, which can be associated with symptoms such as weakness or pain. In a minority of patients, rhabdomyolysis occurs, which is characterized by a massive increase of serum creatine kinase activity and appearance of myoglobin in the urine, which can impair renal function due to precipitation in the tubules^3–5^.

While the clinical picture of statin-associated myopathy has been described well, the mechanisms of statin-associated myopathy are still a matter of debate. From a clinical standpoint, the most important risk factor is increased exposure to statins. This is illustrated by a dose-dependency of statin-associated creatine kinase (CK) elevation and symptoms of myopathy^6^, by drug interactions increasing the systemic statin concentration^5^ and by genetic polymorphisms associated with a decrease in the activity of the organic-anion-transporting polypeptide 1B1 (OATP1B1), which transports statins into hepatocytes^7^. Regarding the molecular mechanisms, by which statins affect skeletal muscle, several possibilities have been proposed. Possible mechanisms include, among others, impairment of mitochondrial function^8,9^, increase of skeletal muscle breakdown due to raised expression of atrogin-1 (MAFbx)^10^, reduction of skeletal muscle protein synthesis^11^, inhibition of small GTPases due to impaired prenylation^12^ and/or impaired creatine synthesis^13^.

**Figure 1:**
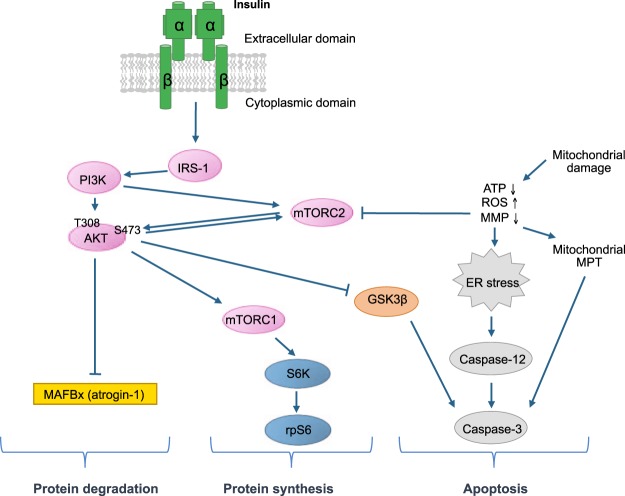
Simplified representation of the IR/Akt/mTOR and related pathways. Upon binding of insulin to its receptor (IR), autophosphorylation and activation of the receptor occurs, leading to the translocation of Akt to the plasma membrane where it is phosphorylated at the Thr308 site by PI3K and at the Ser473 site by mTORC2. After full activation, Akt promotes protein synthesis via mTORC1 activation and prevents caspase activation by phosphorylating and thereby inhibiting glycogen synthase kinase (GSK) 3β. Activated Akt also inhibits protein degradation by repressing MAFBx mRNA expression. Mitochondrial damage is associated with a drop in the cellular ATP content, reactive oxygen species (ROS) production and a drop in the mitochondrial membrane potential (MMP). This leads to impaired activation of mTORC2 and activation of apoptosis via mitochondrial membrane permeability transition (MPT) and ER stress. While insulin inhibits apoptosis by activation of Akt, it can also increase ER stress in the presence of ER stress inducers and thereby stimulate cleavage of caspase-12.

We have reported repeatedly that statins suppress the activation of Akt in a concentration-dependent manner mainly by impairing the phosphorylation of serine 473 (Ser473)^14–17^. Akt is an important protein kinase located in the insulin receptor and insulin-like growth factor (IGF-1) receptor signaling pathway, which for instance phosphorylates and thereby inhibits tuberous sclerosis complex 2 (TSC2) and glycogen synthase kinase 3β (GSK3β) (Fig. 1)^18,19^. Inhibition of TSC2 is associated with activation of mTORC1, which phosphorylates and activates S6 kinase (S6K) and S6 ribosomal protein (rp6S), thereby stimulating protein synthesis^18,19^. Inhibition of GSK3β impairs activation of caspase-3, thereby inhibiting apoptosis^20^. In addition, Akt phosphorylates FoxO3, which cannot reach the nucleus in the phosphorylated form and can therefore not stimulate the transcription of atrogin-1 (MAFbx). Atrogin-1 encodes an ubiquitin ligase associated with muscle atrophy^21,22^. A comparison of the effects of the insulin receptor/Akt signaling pathway with the proposed mechanisms of simvastatin-associated myopathy shows that many of the proposed mechanisms can be explained by the inhibition of Akt.

In a previous publication, we have shown that IGF-1 is able to prevent the toxicity of simvastatin on C2C12 myotubes^16^. Since insulin uses the same intracellular signaling pathway than IGF-1, we were interested whether this is also true for insulin. This appears to be important for several reasons. First, it would emphasize and prove the importance of Akt activation in statin-associated myotoxicity, since Akt plays a central role in both signaling pathways. Second, patients treated with statins can develop insulin resistance and diabetes^23,24^. If insulin were able to prevent or even restore the effect of simvastatin on Akt phosphorylation, this could give an explanation regarding the mechanisms of insulin resistance associated with statins. We therefore decided to investigate the effects of insulin on simvastatin-associated toxicity on C2C12 myotubes and on the insulin receptor signaling pathway. The study shows that insulin cannot only prevent, but also restore, simvastatin-associated toxicity on C2C12 myotubes and can prevent impaired function of Akt and associated downstream events.

## Results

### Simvastatin induced cytotoxicity on C2C12 myotubes, which could be prevented by insulin

**Figure 2:**
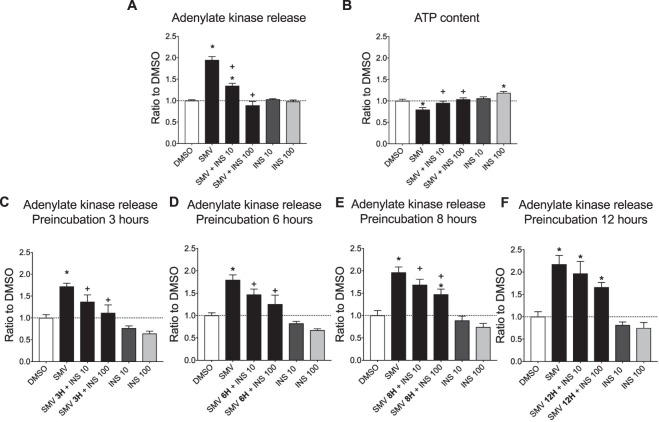
Insulin prevented and reversed the cytotoxicity induced by simvastatin after 24 hours exposure in C2C12 myotubes. (**A**) Cytotoxicity induced in C2C12 myotubes after exposure for 24 hours with 10 μM simvastatin and/or 10 or 100 ng/mL insulin. (**B**) Cellular ATP content in myotubes after 24 hours incubation with simvastatin and/or insulin. (**C**–**F**) Cells were first incubated with simvastatin and then the two concentrations of insulin were added 3 (**C**), 6 (**D**), 8 (**E**) or 12 (**F**) hours later. Data represent the mean ± SEM of four independent experiments. *P < 0.05 *versus* 0.1% DMSO; ^+^P < 0.05 *versus* 10 μM simvastatin. SMV: simvastatin, INS: insulin.

We first investigated adenylate kinase (AK) release (plasma membrane integrity) and ATP content in C2C12 myotubes exposed to simvastatin 10 μM and/or insulin at 10 and 100 ng/mL for 24 h. As expected, the positive control (Triton X 1%) showed an increase of adenylate kinase in the cell supernatant and a decrease in the ATP content (data not shown). In the presence of simvastatin, we observed a 2-fold increase in AK release and a significant drop of intracellular ATP, whereas insulin alone at 10 and 100 ng/mL was not cytotoxic (Fig. 2A,B). However, we could note that the ATP content increased significantly with 100 ng/mL insulin alone compared to control conditions (Fig. 2B). The addition of insulin to 10 μM simvastatin prevented AK release and ATP depletion by simvastatin in a concentration-dependent manner (Fig. 2A,B). In order to investigate whether insulin could not only prevent, but also reverse the simvastatin-associated membrane toxicity, cells were first exposed to simvastatin for 3, 6, 8 and 12 hours prior the addition of insulin at both indicated concentrations. Insulin 10 ng/mL could reverse membrane toxicity after an incubation with simvastatin for 3 hours, and insulin 100 ng/mL after an incubation with simvastatin for 8 hours (Fig. 2C–F).

### Simvastatin treatment increased the synthesis and processing of the insulin receptor and activated procaspase-12 in the endoplasmic reticulum

**Figure 3:**
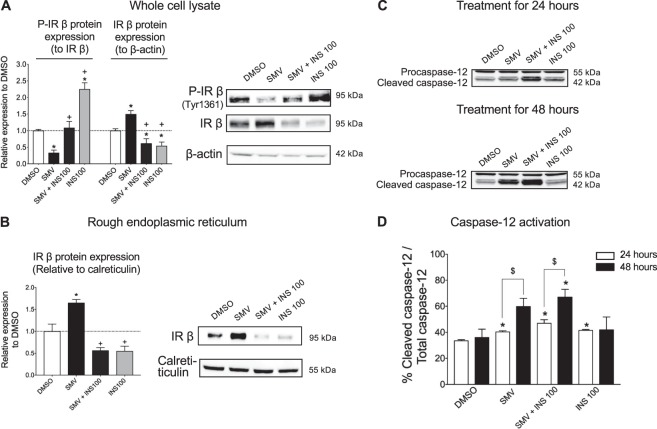
Simvastatin increased protein expression of the insulin receptor β-chain, but impaired its phosphorylation, and induced endoplasmic reticulum stress in C2C12 myotubes. (**A**) Quantification of the phosphorylation and total protein expression of the insulin receptor β-chain in the whole cells and corresponding Western blots. β-actin expression was used for standardization. (**B**) Quantification of the insulin receptor β-chain expression in the rough endoplasmic reticulum and corresponding Western blots. Calreticulin expression was used for standardization and organelle specificity. (**C**) Immunoblots showing the full and cleaved forms of the caspase-12 in myotubes treated for 24 and 48 hours. (**D**) Quantification of the caspase-12 activation. The groups of images were cropped from different blots. Full-length blots are presented in Supplementary Fig. 1. Data represent the mean ± SEM of three independent experiments. *P < 0.05 versus 0.1%DMSO; ^+^P < 0.05 versus 10 μM simvastatin; ^$^P < 0.05 48 hours versus 24 hours. SMV: simvastatin, INS: insulin.

In order to explore the toxicity of simvastatin on C2C12 myotubes and the prevention by insulin, we first focused on the insulin receptor. In cell lysates, simvastatin significantly decreased the phosphorylation of the insulin receptor β (Fig. 3A), while insulin alone or in co-treatment stimulated and prevented the phosphorylation (Fig. 3A). In addition, protein expression of the β-subunit of the insulin receptor was increased by 10 µM simvastatin (Fig. 3A). In contrast, 100 ng/mL insulin decreased the expression of the insulin receptor and prevented the increase in the presence of simvastatin. Subsequently, we separated the endoplasmic reticulum (ER) and analyzed the expression of the insulin receptor. Again, simvastatin alone increased the expression of the insulin receptor and insulin prevented this increase (Fig. 3B). Accumulation of proteins in the ER due to various insults is known to induce ER stress^25^. We evaluated the induction of ER stress in cells by Western blot analysis using an anti-caspase-12 antibody that recognizes the cleaved and the pro forms of this caspase (Fig. 3C). After 24 hours of exposure, simvastatin alone increased cleavage of procaspase-12, indicating ER stress (Fig. 3D). An increase in procaspase-12 was also observed in the presence of insulin and was accentuated for the combination of simvastatin and insulin. After 48 h of exposure, we found that the expression of the cleaved form of caspase-12 was stable in the control (DMSO) and insulin samples compared to exposure for 24 hours. However, in the presence of simvastatin or the combination of simvastatin and insulin, cleavage of procaspase-12 was significantly increased compared to incubation for 24 hours (Fig. 3C,D). These findings suggested that simvastatin induced ER stress by retaining proteins such as the insulin receptor in the ER and that insulin increased the ER stress in the presence of simvastatin despite suppressing the synthesis of the insulin receptor β-chain.

### Insulin prevented the impairment of protein synthesis and atrogin-1 expression in C2C12 myotubes treated with simvastatin

**Figure 4:**
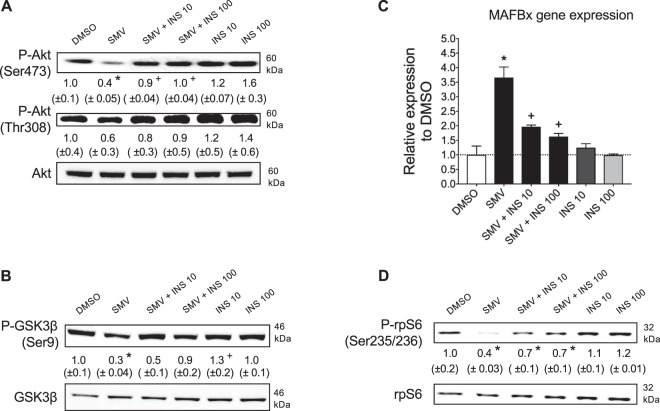
Insulin prevented the impairment of Akt, GSK3β and S6 ribosomal protein phosphorylation and the increase in MAFbx mRNA expression in C2C12 myotubes by simvastatin. (**A**) Immunoblots showing two phosphorylated forms of the kinase Akt (Ser473 and Thr308) and its total protein expression. (**B**) Immunoblots of the phosphorylated (Ser9) and total GSK3β. (**C**) mRNA expression of MAFbx in C2C12 myotubes determined by real-time PCR. (**D**) Representative immunoblots of the phosphorylated S6 rp (Ser235/236) compared to its total form. The groups of images were cropped from different blots. Full-length blots are presented in Supplementary Fig. 2. Data represent the mean ± SEM of three independent experiments. *P < 0.05 versus 0.1% DMSO. ^+^P < 0.05 versus 10 μM simvastatin. GSK3β: Glycogen synthase kinase 3β; MAFbx: Muscle atrophy F-Box; rp: ribosomal protein.

Next, we investigated the effects of simvastatin and insulin on the insulin receptor signaling pathway (Fig. 1). As previously reported^16^, 10 µM simvastatin was associated with a significant decrease in Akt phosphorylation at the Ser473, whereas the Thr308 phosphorylation was decreased by trend only (Fig. 4A). Co-treatment with insulin restored the phosphorylation of Akt at both phosphorylation sites at both concentrations used (Fig. 4A). Activation of Akt indirectly activates mTORC1, which phosphorylates S6K. S6K phosphorylates and thereby activates the ribosomal protein S6 (rpS6), which promotes mRNA translation and thus protein synthesis (Fig. 1). In order to investigate the activity of Akt, we first evaluated the Ser9 phosphorylation of GSK3β. As shown in Fig. 4B, simvastatin decreased the phosphorylation of GSK3β at the Ser9, which is associated with stimulation of caspases and apoptosis^26^. Importantly, insulin partially restored the phosphorylation of GSK3β at Ser9. Next, we investigated the mRNA expression of MAFbx, which encodes for atrogin-1, an ubiquitin ligase associated with muscle atrophy whose expression is suppressed by Akt (Fig. 1). While insulin alone did not affect the gene expression of MAFbx (Fig. 4C), simvastatin significantly increased MAFbx mRNA expression. The addition of insulin at least partially prevented this increase at both concentrations used. Finally, as expected from the decreased activation of Akt, we found a significant decrease in rpS6 phosphorylation with simvastatin, suggesting decreased protein synthesis (Fig. 4D). Co-treatment with insulin at both concentrations partially prevented the phosphorylation status of rpS6.

### Simvastatin activated the apoptotic cascade and PARP in C2C12 myotubes

**Figure 5:**
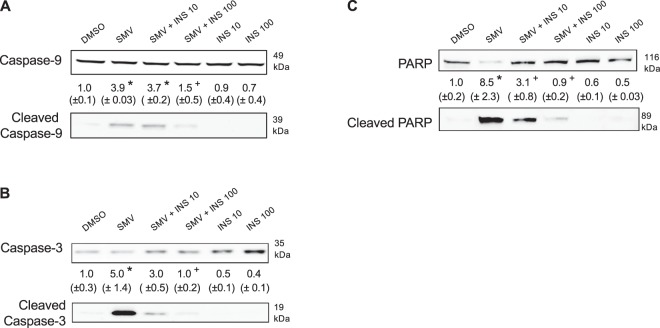
Simvastatin induced the activation of apoptotic caspases in C2C12 cells, which was prevented by insulin. (**A**) Immunoblots of the full and cleaved caspase-9. (**B**) Representative immunoblots of the full and cleaved caspase-3. C. Representative immunoblots of the full and cleaved PARP. The groups of images were cropped from different blots. Full-length blots are presented in Supplementary Fig. 3. Data represent the mean ± SEM of three independent experiments. *P < 0.05 *versus* 0.1% DMSO. ^+^P < 0.05 *versus* 10 μM simvastatin. PARP: Poly (ADP-ribose) polymerase.

As shown in Fig. 1, activation of Akt inhibits protein degradation, stimulates protein synthesis and impairs apoptosis. After having shown that insulin could prevent the simvastatin-associated drop in the cellular ATP content and GSK3β phosphorylation, we also investigated the effect simvastatin and insulin on apoptosis. Simvastatin induced a strong cleavage of the initiator caspase-9 and the executioner caspase-3, indicating the activation of the apoptotic cascade in myotubes (Fig. 5A,B). Insulin partially (10 ng/mL) or completely (100 ng/mL) inhibited the activation of these caspases by simvastatin. In addition, we analyzed the activation of PARP, a protein engaged in DNA repair, which is cleaved by caspase-3 during apoptosis^27^. As shown in Fig. 5C, simvastatin was associated with a strong cleavage of PARP, which could partially (10 ng/mL) or completely (100 ng/mL) be inhibited by the addition of insulin to simvastatin.

### Insulin prevented cell death associated with simvastatin but not with the Akt inhibitor MK-2206

**Figure 6:**
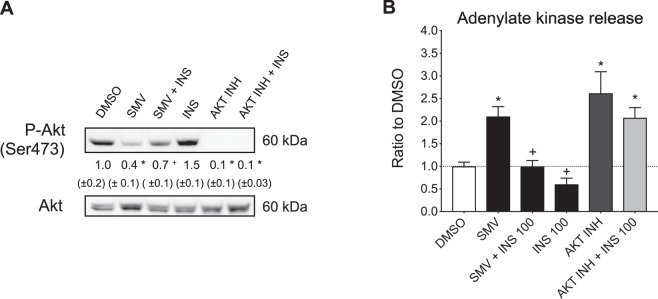
Insulin prevented impairment of Akt Ser473 phosphorylation and cell death by simvastatin, but not by MK-2206. C2C12 myotubes were exposed for 24 hours with 10 μM simvastatin and/or 100 ng/mL insulin. Myotubes were also treated with 10 μM MK-2206, an allosteric pan-Akt inhibitor, alone or together with 100 ng/mL insulin. (**A**) Quantification of the phosphorylation (Ser473) and total protein expression of Akt and corresponding Western blots. (**B**) Cytotoxicity determined as the release of adenylate kinase. Data represent the mean ± SEM of three independent experiments. *P < 0.05 versus 0.1% DMSO; ^+^P < 0.05 versus 10 μM simvastatin. SMV: simvastatin, INS: insulin, AKT INH: MK-2006, pan-Akt inhibitor.

In order to demonstrate the role of impaired activation of Akt for cytotoxicity in more detail, we performed a comparison of the effect of insulin on C2C12 myotubes treated with simvastatin and with the allosteric pan-Akt inhibitor MK-2206. As shown in Fig. 6A, MK-2206 blocked Akt phosphorylation at Ser473 almost completely, irrespectively of the presence of insulin. In comparison, simvastatin inhibited Akt Ser473 phosphorylation only partially and this block could almost completely be prevented by insulin. Accordingly, insulin was not able to prevent cell death associated with MK-2206, while insulin prevented cell death associated with simvastatin almost completely. The experiment shows the important role of Akt activation for the prevention of cell death and suggests that the effect of simvastatin on Akt is not direct and can be circumvented by insulin signaling.

## Discussion

The study demonstrates that 10 µM simvastatin was cytotoxic for C2C12 myotubes and impaired the insulin signaling pathway, leading to a reduced activation of Akt. Reduced activation of Akt was associated with increased mRNA expression of MAFBx, reduced activity of mTORC1 and induction of apoptosis. Insulin at physiological (10 ng/mL) or supraphysiological concentrations (100 ng/mL) could at least partially prevent or even revert these changes.

As already described previously^15,16^, simvastatin mainly impaired the phosphorylation of Ser473 in Akt, which is dependent on the activity of mTORC2^18,19^. This suggests that the primary insult of simvastatin is the reduction of the activity of mTORC2. This assumption is supported by the observation that insulin could prevent cell death in the presence of simvastatin, but not in the presence of the pan-Akt inhibitor MK-2206. In comparison to mTORC1, the activation of mTORC2 is less well investigated^26^. Inactivation of mTORC2 due to impaired assembly of the mTORC2 complex has been described as a consequence of oxidative damage by reactive oxygen species^28^. As shown in the current and in previous studies^14,15^, simvastatin reduces the cellular ATP content, suggesting mitochondrial dysfunction. In support of this assumption, we and others have shown previously that simvastatin and other statins can impair mitochondrial function^8,9,29,30^, for instance by inhibiting the electron transport chain. Since inhibition of complex I and III of the electron transport chain is associated with increased ROS production^31^, this represents a possible mechanism how simvastatin can reduce the activity of mTORC2. In support of this hypothesis, we have shown previously in cell cultures and skeletal muscle from experimental animals and humans that statins are associated with increased ROS production and oxidative damage in mainly glycolytic skeletal muscle^8,29,32^.

Activation of mTORC2 has also been described to be dependent on insulin/phosphoinositide kinase (PI3K) signaling^26^. A link between the insulin/PI3K pathway and mTORC2 is given by mSin1, which is a subunit of mTORC2 inhibiting mTORC2 activation. The inhibition of mTORC2 activation by mSin1 can be relieved by binding of mSin1 to PI3K-generated PIP_3_ in the plasma membrane^33^ or by phosphorylation by Akt, suggesting the existence of a positive feedback loop between mTORC2 and Akt (see Fig. 1)^34^. The inhibition of mTORC2 activation by simvastatin could therefore also be explained by an impairment of the insulin/PI3K pathway. Indeed, simvastatin impaired by trend the phosphorylation of Akt Thr308, which depends on insulin/PI3K signaling (Fig. 4A). Importantly, the addition of insulin partially prevented the impairment of Akt Thr308 phosphorylation by simvastatin, indicating that the impairment of the insulin/PI3K pathway by simvastatin can be overcome by strong receptor stimulation. This is in agreement with our previous report showing that impaired phosphorylation of Akt by simvastatin can be partially prevented by stimulation of the IGF-1 receptor^16^.

Finally, since statins block 3-hydroxy-3-methylglutaryl-CoA also in skeletal muscle^35^, treatment with statins is associated with a decrease of intermediates in cholesterol biosynthesis such as farnesyl- and geranylgeranylpyrophosphate in skeletal muscle, which can impair the prenylation and therefore the correct location and function of membrane-bound proteins. Whether there is a connection between impaired protein prenylation and reduced activation of mTORC2 by simvastatin has, to the best of our knowledge, so far not been shown and should be investigated in future studies. In support of the hypothesis that protein prenylation could be involved in impaired activation of mTORC2, the addition of mevalonate has been shown to partially prevent certain aspects of the toxicity of statins on skeletal muscle cells^36^.

Based on the results of the current study, the promotion of apoptosis by simvastatin can be explained by three mechanisms. Firstly, simvastatin was associated with activation of caspase-12, suggesting an unfolded protein response or ER stress^37^. Secondly, by inhibiting the full activation of Akt, simvastatin impaired the phosphorylation of GSK3β (see Fig. 1), leading to a stimulation of caspase-3 and apoptosis. Thirdly, simvastatin caused mitochondrial damage (as suggested by the decrease in the cellular ATP), which is associated with apoptosis^8^. The association of simvastatin with ER stress has been described previously. For instance, Ghavami *et al*. described simvastatin-associated ER stress in human atrial fibroblasts^38^ and Mörck *et al*. in *C. elegans*^39^. Interestingly, Mörck *et al*. showed that farnesyl pyrophosphate was able to prevent fluvastatin-associated ER stress in *C. elegans*, whereas geranylgeranyl pyrophosphate was ineffective. This suggested that fluvastatin inhibits the prenylation of RAS peptides, which was proven by knock-out experiments. Prenylated RAS peptides appear therefore to be important regulators of the protein folding mechanism in the ER. In the current study, in contrast to cytotoxicity and apoptosis, insulin stimulated the effect of simvastatin on caspase-12 activation, suggesting stimulation, rather than prevention, of ER stress. This may be due to the activation of mTORC1 by insulin via the insulin receptor/PI3K pathway, which is associated with an increase in protein synthesis^26^. As shown in the current study, the addition of insulin improved the activity of Akt in the presence of simvastatin, leading to increased phosphorylation of GSK3β, which inhibits apoptosis^26^. Furthermore, the addition of insulin improved mitochondrial function (as suggested by the normalization of the cellular ATP content), which also should reduce apoptosis. The positive effect of insulin on mitochondrial function has been demonstrated previously in other cell models^40^. The inhibition of apoptosis by insulin in the presence of simvastatin is therefore most likely a consequence of increased insulin signaling and improved mitochondrial function, which predominate the pro-apoptotic effect associated with caspase 12 stimulation.

It is well established that patients treated with statins can develop insulin resistance^23,24^. Our study is compatible with this observation and suggests that this may be due to impaired insulin signaling and/or impaired activity of mTORC2. Recently, Sun *et al*. reported that simvastatin impairs the translocation of insulin-responsible glucose transporter 4 (GLUT4) from the ER to the plasma membrane in C2C12 myotubes due to a decrease in the cellular cholesterol content^41^. In addition, Kleinert *et al*. published that mTORC2 inhibition was associated with impaired glucose uptake and metabolism by muscle cells due to impaired glycolysis^42^. Taking into account the findings of the current study, ER stress and impaired activation of Akt and mTORC2 could be possible reasons for reduced uptake of glucose by myotubes and skeletal muscle in the presence of statins. ER stress could impair the translocation of GLUT4 from the ER to the plasma membrane by retaining proteins in the ER and Akt activation has been shown to be essential for GLUT4 translocation^20^ and, as discussed above, also for activation of mTORC2^26^. Taking into account the clinical observation that treatment with insulin is able to overcome statin-associated insulin resistance and the results of the current study, impaired activation of Akt appears to be the more likely reason for insulin resistance than ER stress. In the current study, insulin improved the activation of Akt whereas it accentuated ER stress associated with simvastatin.

The current study has also some deficiencies. For instance, we did not show the effect of simvastatin on the insulin-signaling pathway between the insulin receptor and Akt. Since the phosphorylation of both the insulin receptor and Akt Thr308 was impaired, we assume that this was also the case for the intermediates (see Fig. 1). Furthermore, we investigated the effects of simvastatin and insulin only in C2C12 myotubes and not in other cell lines or in skeletal muscle from animals or humans. We have shown previously that simvastatin impairs Akt activation in skeletal muscle of mice^15^ and that statins are toxic in skeletal muscle biopsies from humans^32^. We therefore assume to find similar effects of insulin on simvastatin-associated myotoxicity also in animals and humans.

In conclusion, simvastatin impaired the phosphorylation of Akt at Ser473 due to reduced activity of mTORC2. Impaired activation of Akt caused increased mRNA expression of atrogin-1, decreased activation of mTORC1 and induced apoptosis. Furthermore, simvastatin was associated with ER stress. Insulin prevented impaired activation of Akt S473 concentration-dependently but stimulated ER stress. Impaired activation of mTORC2 appears to be a key event for simvastatin-associated toxicity on C2C12 myotubes, which deserves further investigations.

## Methods

### Chemicals

Simvastatin lactone was obtained from Sigma-Aldrich (St-Louis, USA) and converted to the acid form by hydrolysis^43^. Stock solutions were prepared in DMSO (Sigma-Aldrich, USA) and stored at −20 °C. Human insulin was purchased from Sigma-Aldrich and stored at 4 °C. Stock solutions were prepared in culture medium. Akt inhibitor MK-2206 was purchased from Santa Cruz Biotechnology (USA) and stocks solutions were prepared in DMSO.

### Cell line and maintenance

C2C12 myoblasts (American Type Culture Collection, USA) had been provided by Novartis (Basel, Switzerland). Myoblasts were cultured in Dulbecco’s Modified Eagle Medium (DMEM) containing GlutaMAX, which was supplemented with 10% fetal bovine serum (FBS) and 1% HEPES (Gibco, UK). Cells were kept at 37 °C in a humidified cell culture incubator and exposed to 5% CO_2_. They were passaged using trypsin upon reaching approximately 60% confluence and seeded in appropriate well plates prior differentiation into myotubes. Two days after seeding, the medium was replaced by differentiation medium (containing DMEM-GlutaMAX, 1% HEPES and 2% horse serum [Gibco, UK]) for three days. The resulting myotubes were then treated with simvastatin and/or insulin in serum-free differentiation medium.

### Membrane toxicity

Myoblasts were seeded in a 96-well plate at 5,000 cells per well and differentiated to myotubes. We used the ToxiLight^TM^ (Lonza, Basel, Switzerland) assay to assess the cytotoxicity of 10 μM simvastatin, 10 µM MK-2206 (Akt inhibitor) and/or 10 ng/mL and 100 ng/mL insulin 24 hours after the treatment. For rescue experiments, myotubes were first treated with simvastatin for 3, 6, 8 and 12 hours before insulin was added. We used Triton-X 1% as a positive control, which destroys cells completely, resulting in a 4-fold increase in adenylate kinase release approximately. The release of adenylate kinase (AK) was measured according to the manufacturer’s instructions. Briefly, ToxiLight^TM^ reaction buffer (100 μL) was mixed with 20 μL of supernatant from the treated cells. Then, the mixture was left for 5 minutes and the luminescence was measured with a Tecan M200 Pro Infinity plate reader (Männerdorf, Switzerland).

### ATP content

Myoblasts (5,000 cells/well) were seeded in a 96-well plate and differentiated to myotubes. Myotubes were then exposed to 10 μM simvastatin and/or 10 ng/mL or 100 ng/mL insulin for 24 hours. We used Triton-X 1% as a positive control, which destroys cells and depletes the intracellular ATP content completely. Intracellular ATP was determined using the CellTiterGlo Luminescent cell viability assay (Promega, Switzerland), in accordance with the manufacturer’s instructions. Briefly, 100 μL of assay buffer was added to each 96-well containing 100 μL culture medium. After incubation for 10 minutes, luminescence was measured using a Tecan M200 Pro Infinity plate reader (Männerdorf, Switzerland).

### Isolation of membranes of the endoplasmic reticulum

Rough endoplasmic reticulum membranes were isolated using the Endoplasmic Reticulum Enrichment Kit (Novus Biologicals, USA) according to the manufacturer’s instructions. Western blot analysis for endoplasmic reticulum specific and other marker proteins was performed to assess the purity of the isolated membranes.

### Real-time PCR

RNA extraction from cells was performed using the Qiagen RNeasy mini extraction kit (Qiagen, Switzerland) according to the manufacturer’s instructions and as described earlier^44^. The RNA concentration and purity of each mRNA sample was determined using the NanoDrop 2000 (Thermo Scientific, Switzerland). cDNA was obtained from 1 μg RNA using the Qiagen Omniscript system (Qiagen, Switzerland). Amplification reactions were performed using SYBR green (Roche Diagnostics, Switzerland) and specific forward and reverse primers.

The following primers were used: atrogin-1 (MAFbx) forward 5′-AGTGAGGACCGGCTACTGTG-3′ and reverse 5′-GATCAAACGCTTGCGAATCT-3′; GAPDH forward 5′-CATGGCCTTCCGTGTTCCTA-3′ and reverse 5′-CCTGCTTCACCACCTTCTTGA-3′.

Real time PCR was performed using the ViiA7 software (Life Technologies, Switzerland) on an ABI PRISM 7700 sequence detector (PE Biosystems, Switzerland). The ΔΔCt method was used to determine relative gene expression levels and the values were normalized to the housekeeping gene (GAPDH).

### Western blots

Western blots were prepared as described previously^44^. In brief, cells were grown and differentiated on 6-well culture plates and treated with the compounds of interest for 24 hours. After treatment, they were washed twice with cold PBS (Gibco, UK) and lysed in Phosphosafe buffer (EMD Millipore, USA) for 5 minutes on ice. The resulting cell lysates were centrifuged at 1,600 g for 10 minutes at 4 °C. The supernatants were collected and the protein content was determined using the BCA Protein Assay kit (Pierce, Thermo Scientific, USA). After dilution with lithium dodecyl sulfate (LDS) sample buffer (Invitrogen, Switzerland) and heating at 93 °C for 5 minutes, proteins were separated on NuPAGE^TM^ 4–12% Bis-Tris polyacrylamide gels (Invitrogen, Switzerland) at 140 volts. Gels were then transferred to polyvinylidendifluoride membranes (Bio-Rad Laboratories, USA). After protein transfer, membranes were incubated for 1 hour in 5% non-fat dry milk in PBS containing 0.1% Tween-20 (Sigma-Aldrich, USA) blocking solution. Then, membranes were incubated overnight with the following primary antibodies diluted 1:1000 in the blocking solution: phospho-insulin receptor β (Tyr1361) and insulin receptor β (Cell Signaling Technology, USA), calreticulin (Abcam, UK), caspase-12 for full and cleaved forms (Cell Signaling Technology, USA), phospho-Akt (Ser473 and Thr308), Akt (Cell Signaling Technology, USA), phospho-S6 ribosomal protein (Ser235/236), S6 ribosomal protein (Cell Signaling Technology, USA), phospho-GSK3β (Ser9) and GSK3β (Cell Signaling Technology, USA), caspase-3 for full and cleaved forms (Cell Signaling Technology, USA), caspase-9 for full and cleaved forms (Cell Signaling Technology, USA) and PARP for full and cleaved products (Cell Signaling Technology, USA). GAPDH (Santa Cruz Biotechnology, USA) and beta-actin (Abcam, UK) were diluted 1:6000. Secondary antibodies (Santa Cruz Biotechnology, USA) were used for 1 hour diluted 1:2000 in the blocking solution. Membranes were then washed and protein bands were developed using the Clarity^TM^ Western ECL Substrate (Bio-Rad Laboratories, USA). Protein expression was quantified using the Fusion Pulse TS device from Vilber Lourmat (Oberschwaben, Germany). Equal loading of the samples was checked using the quantity of housekeeping genes beta-actin or GAPDH.

### Statistical analysis

Results are presented as mean ± SEM. Data were analyzed by unpaired Student’s t test (comparison of two groups) or one-way ANOVA with Newman-Keuls’s post-hoc test (comparison of multiple groups) using GraphPad Prism 7 (GraphPad Software, La Jolla, CA, US). Differences between groups were considered to be significant at p < 0.05.

## Supplementary information

Supplementary legends and western blots

## References

[1] Sirtori CR (2014). The pharmacology of statins. Pharmacological research: the official journal of the Italian Pharmacological Society.

[2] Baigent C (2010). Efficacy and safety of more intensive lowering of LDL cholesterol: a meta-analysis of data from 170,000 participants in 26 randomised trials. Lancet (London, England).

[3] Armitage J (2007). The safety of statins in clinical practice. Lancet (London, England).

[4] Alfirevic A (2014). Phenotype standardization for statin-induced myotoxicity. Clinical pharmacology and therapeutics.

[5] Roten L, Schoenenberger RA, Krahenbuhl S, Schlienger RG (2004). Rhabdomyolysis in association with simvastatin and amiodarone. The Annals of pharmacotherapy.

[6] Joy TR, Hegele RA (2009). Narrative review: statin-related myopathy. Annals of internal medicine.

[7] Link E (2008). SLCO1B1 variants and statin-induced myopathy–a genomewide study. The New England journal of medicine.

[8] Kaufmann P (2006). Toxicity of statins on rat skeletal muscle mitochondria. Cellular and Molecular Life Sciences.

[9] Kwak HB (2012). Simvastatin impairs ADP-stimulated respiration and increases mitochondrial oxidative stress in primary human skeletal myotubes. Free radical biology & medicine.

[10] Hanai J (2007). The muscle-specific ubiquitin ligase atrogin-1/MAFbx mediates statin-induced muscle toxicity. The Journal of clinical investigation.

[11] Tuckow AP, Jefferson SJ, Kimball SR, Jefferson LS (2011). Simvastatin represses protein synthesis in the muscle-derived C(2)C(1)(2) cell line with a concomitant reduction in eukaryotic initiation factor 2B expression. American journal of physiology. Endocrinology and metabolism.

[12] Sakamoto K, Wada I, Kimura J (2011). Inhibition of Rab1 GTPase and endoplasmic reticulum-to-Golgi trafficking underlies statin’s toxicity in rat skeletal myofibers. The Journal of pharmacology and experimental therapeutics.

[13] Mangravite LM (2013). A statin-dependent QTL for GATM expression is associated with statin-induced myopathy. Nature.

[14] Bonifacio A (2016). Simvastatin induces mitochondrial dysfunction and increased atrogin-1 expression in H9c2 cardiomyocytes and mice *in vivo*. Archives of toxicology.

[15] Bonifacio A, Sanvee GM, Bouitbir J, Krahenbuhl S (2015). The AKT/mTOR signaling pathway plays a key role in statin-induced myotoxicity. Biochimica et biophysica acta.

[16] Bonifacio A (2017). IGF-1 prevents simvastatin-induced myotoxicity in C2C12 myotubes. Arch Toxicol.

[17] Mullen PJ (2011). Susceptibility to simvastatin-induced toxicity is partly determined by mitochondrial respiration and phosphorylation state of Akt. Biochimica Et Biophysica Acta-Molecular Cell Research.

[18] Laplante M, Sabatini DM (2009). mTOR signaling at a glance. Journal of cell science.

[19] Showkat M, Beigh MA, Andrabi KI (2014). mTOR Signaling in Protein Translation Regulation: Implications in Cancer Genesis and Therapeutic Interventions. Molecular biology international.

[20] Carnagarin R, Dharmarajan AM, Dass CR (2015). Molecular aspects of glucose homeostasis in skeletal muscle–A focus on the molecular mechanisms of insulin resistance. Molecular and cellular endocrinology.

[21] Sandri M (2013). Signalling pathways regulating muscle mass in ageing skeletal muscle: the role of the IGF1-Akt-mTOR-FoxO pathway. Biogerontology.

[22] Stitt TN (2004). The IGF-1/PI3K/Akt pathway prevents expression of muscle atrophy-induced ubiquitin ligases by inhibiting FOXO transcription factors. Mol Cell.

[23] Ridker PM, Pradhan A, MacFadyen JG, Libby P, Glynn RJ (2012). Cardiovascular benefits and diabetes risks of statin therapy in primary prevention: an analysis from the JUPITER trial. Lancet (London, England).

[24] Sattar N (2010). Statins and risk of incident diabetes: a collaborative meta-analysis of randomised statin trials. Lancet (London, England).

[25] Yoshida H (2007). ER stress and diseases. FEBS J.

[26] Saxton RA, Sabatini DM (2017). mTOR Signaling in Growth, Metabolism, and Disease. Cell.

[27] Satoh MS, Lindahl T (1992). Role of poly(ADP-ribose) formation in DNA repair. Nature.

[28] Wang RH (2011). Hepatic Sirt1 deficiency in mice impairs mTorc2/Akt signaling and results in hyperglycemia, oxidative damage, and insulin resistance. The Journal of clinical investigation.

[29] Bouitbir J (2012). Opposite effects of statins on mitochondria of cardiac and skeletal muscles: a ‘mitohormesis’ mechanism involving reactive oxygen species and PGC-1. European heart journal.

[30] Sirvent P (2005). Simvastatin induces impairment in skeletal muscle while heart is protected. Biochemical and biophysical research communications.

[31] Markevich NI, Hoek JB (2015). Computational modeling analysis of mitochondrial superoxide production under varying substrate conditions and upon inhibition of different segments of the electron transport chain. Biochimica et biophysica acta.

[32] Bouitbir J (2016). Statins Trigger Mitochondrial Reactive Oxygen Species-Induced Apoptosis in Glycolytic Skeletal Muscle. Antioxidants & redox signaling.

[33] Liu P (2015). PtdIns(3, 4, 5)P3-Dependent Activation of the mTORC2 Kinase Complex. Cancer Discov.

[34] Yang G, Murashige DS, Humphrey SJ, James DE (2015). A Positive Feedback Loop between Akt and mTORC2 via SIN1 Phosphorylation. Cell Rep.

[35] Mullen PJ, Luscher B, Scharnagl H, Krahenbuhl S, Brecht K (2010). Effect of simvastatin on cholesterol metabolism in C2C12 myotubes and HepG2 cells, and consequences for statin-induced myopathy. Biochemical pharmacology.

[36] Sacher J, Weigl L, Werner M, Szegedi C, Hohenegger M (2005). Delineation of myotoxicity induced by 3-hydroxy-3-methylglutaryl CoA reductase inhibitors in human skeletal muscle cells. The Journal of pharmacology and experimental therapeutics.

[37] Szegezdi E, Fitzgerald U, Samali A (2003). Caspase-12 and ER-stress-mediated apoptosis: the story so far. Annals of the New York Academy of Sciences.

[38] Ghavami S (2012). Apoptosis, autophagy and ER stress in mevalonate cascade inhibition-induced cell death of human atrial fibroblasts. Cell death & disease.

[39] Morck C (2009). Statins inhibit protein lipidation and induce the unfolded protein response in the non-sterol producing nematode Caenorhabditis elegans. Proc Natl Acad Sci USA.

[40] Cheng Z, Tseng Y, White MF (2010). Insulin signaling meets mitochondria in metabolism. Trends Endocrinol Metab.

[41] Sun B (2018). Atorvastatin impaired glucose metabolism in C2C12 cells partly via inhibiting cholesterol-dependent glucose transporter 4 translocation. Biochemical pharmacology.

[42] Kleinert M (2014). Acute mTOR inhibition induces insulin resistance and alters substrate utilization *in vivo*. Molecular metabolism.

[43] Bogman K, Peyer AK, Torok M, Kusters E, Drewe J (2001). HMG-CoA reductase inhibitors and P-glycoprotein modulation. Br J Pharmacol.

[44] Sanvee GM, Panajatovic MV, Bouitbir J, Krahenbuhl S (2019). Mechanisms of insulin resistance by simvastatin in C2C12 myotubes and in mouse skeletal muscle. Biochemical pharmacology.

