# Supplementary material for: Insulin prevents and reverts simvastatin-induced toxicity in C2C12 skeletal muscle cells
Source: Sci Rep. 2019 May 15;9:7409. doi: 10.1038/s41598-019-43938-5 (PMC6520350; doi:10.1038/s41598-019-43938-5)

Insulin prevents and reverts simvastatin-induced toxicity in C2C12 skeletal muscle cells

Running title: Insulin and statin-associated toxicity on myotubes

Gerda M. Sanvee [1,2]Jamal Bouitbir[1,2,3], Stephan Krähenbühl[1,2,3]

[1]Division of Clinical Pharmacology & Toxicology, University Hospital, Basel, Switzerland

[2]Department of Biomedicine, University of Basel, Switzerland

[3]Swiss Centre for Applied Human Toxicology (SCAHT), Basel, Switzerland

Corresponding author

Stephan Krähenbühl, MD, PhD

Clinical Pharmacology & Toxicology

University Hospital

4031 Basel

Switzerland

Phone: +41 61 265 4715

Fax: +41 61 265 4560

E-mail: Stephan.kraehenbuehl@usb.ch

Word count: 3001 words (excluding abstract, methods, references, and figure legends), abstract (241 words), 6 figures, 44 references and 1 supplementary file.

Supplementary Figure 1. Full-length immunoblots of Fig.3.

A. Phospho-insulin receptor β (Tyr1361) in whole cell lysate. **B**. Insulin receptor β in whole cell lysate. **C**. β -actin. **D**. Insulin receptor β in rough endoplasmic reticulum. **E**. Calreticulin. **F**. Full and cleaved caspase-12 after 24 and 48 hours.

Supplementary Figure 2. Full-length immunoblots of Fig.4.

A. Phosphorylated Akt at Ser473. **B**. Phosphorylated Akt at Thr308. **C**. Total Akt. **D**. Phospho-GSK3 β (Ser9). **E**. GSK3 β. **F**. Phosphorylated S6 ribosomal protein at Ser235/236. **G**. Total S6 ribosomal protein.

Supplementary Figure 3. Full-length immunoblots of Fig.5.

A. Full caspase-9. **B**. Cleaved caspase-9. **C**. Full caspase-3. **D**. Cleaved caspase-3. **E**. Full PARP. **F**. Cleaved PARP.

Supplementary Figure 4. Full-length immunoblots of Fig.6.

A. Phosphorylated Akt at Ser473. **B**. Total Akt.

Suppl. Fig. 1

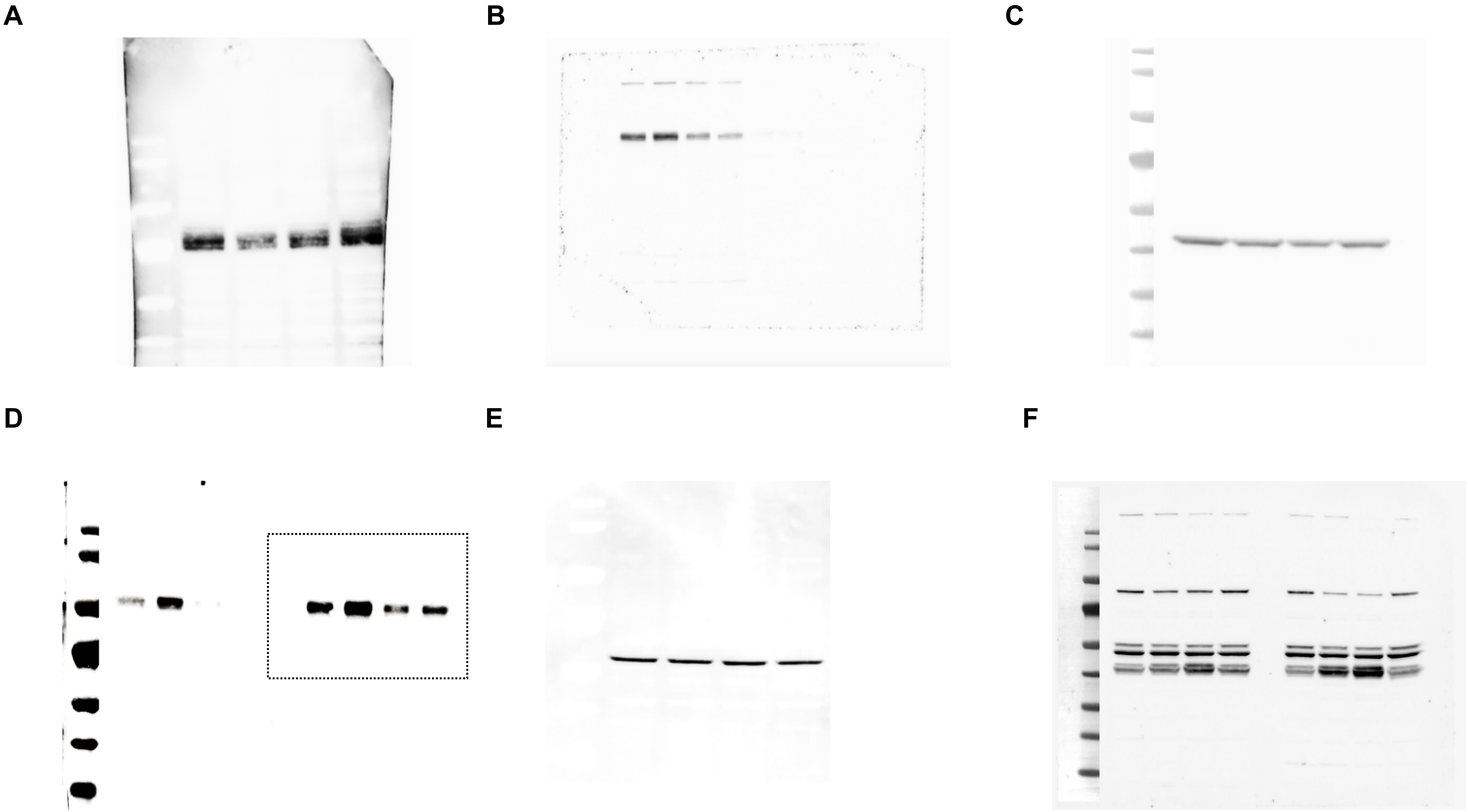

Suppl. Fig. 2

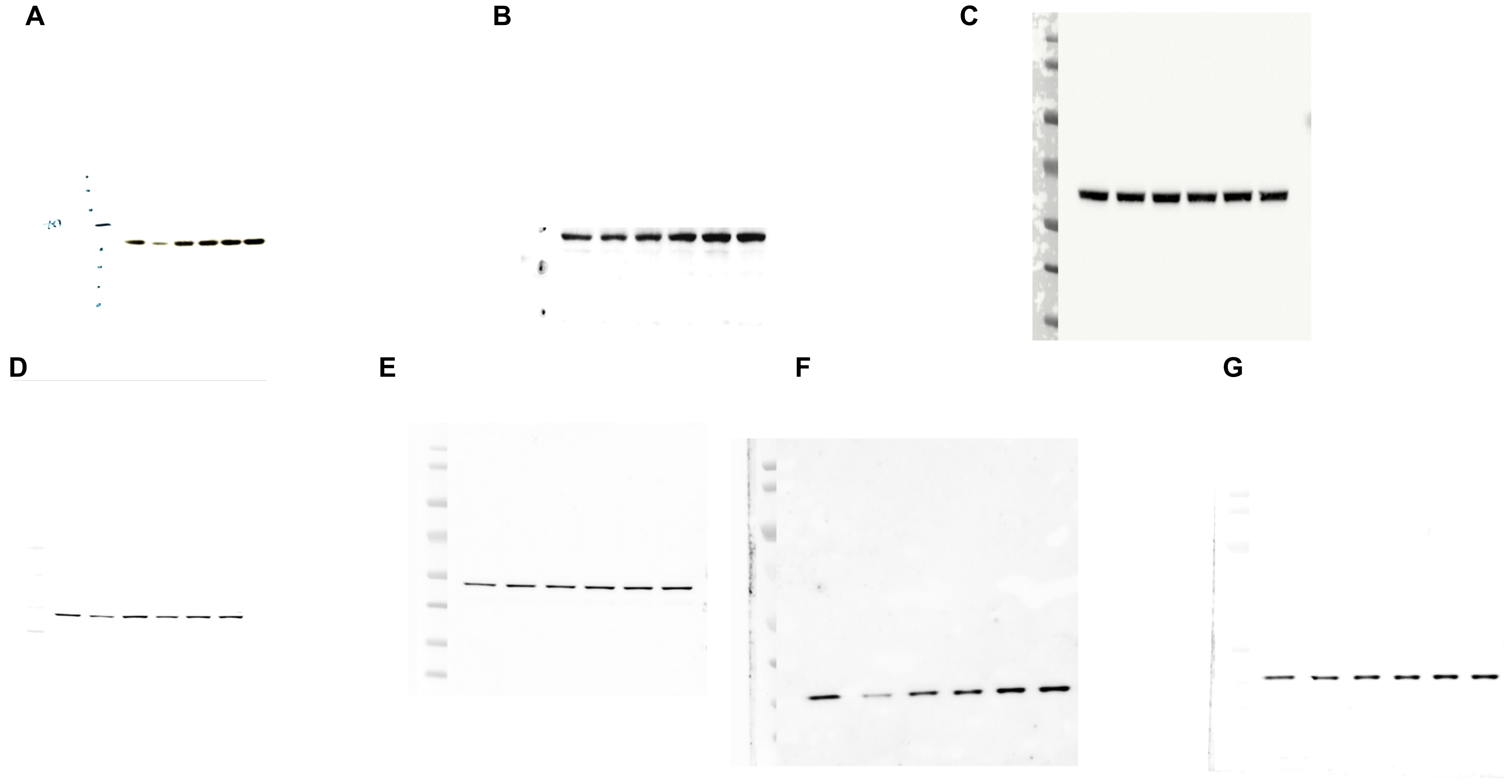

Suppl. Fig. 3

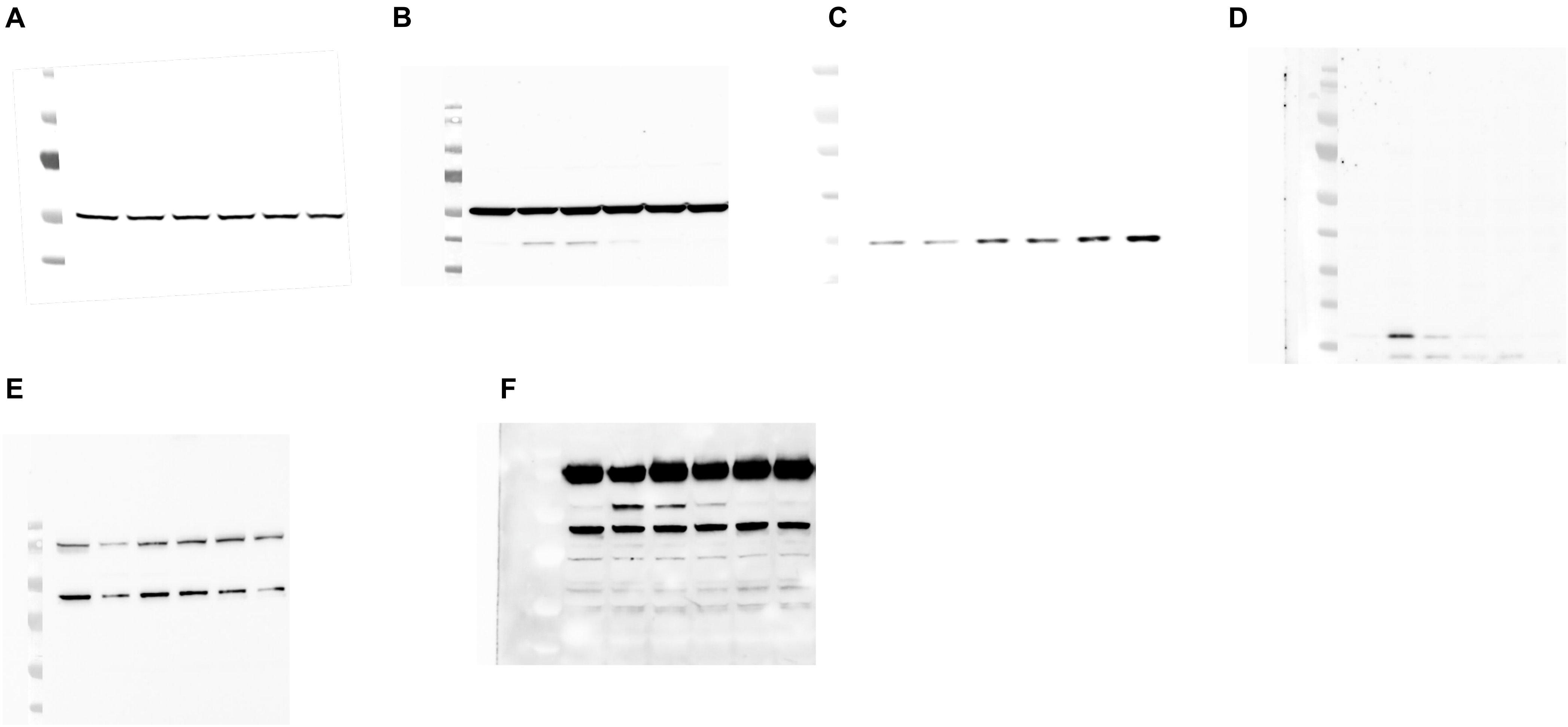

Suppl. Fig. 4

A

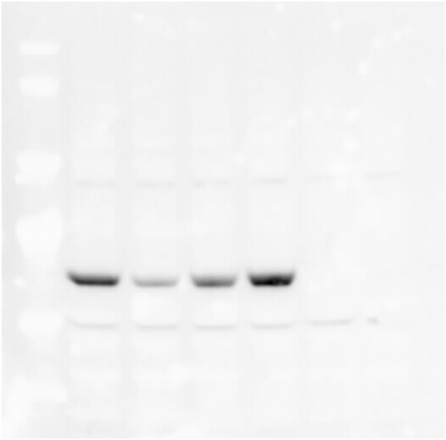

B

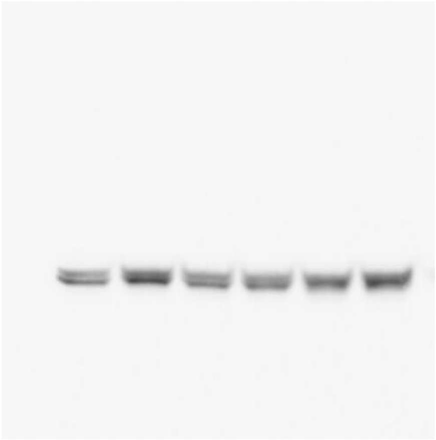

Supplement: Supplementary file 1 — Supplementary legends and western blots [file 41598_2019_43938_MOESM1_ESM.pdf]
